# Differential effects of HIF2α antagonist and HIF2α silencing in renal cancer and sensitivity to repurposed drugs

**DOI:** 10.1186/s12885-021-08616-8

**Published:** 2021-08-05

**Authors:** Esther Arnaiz, Ana Miar, Esther Bridges, Naveen Prasad, Stephanie B. Hatch, Daniel Ebner, Charles H. Lawrie, Adrian L. Harris

**Affiliations:** 1 Department of Medical Oncology, Molecular Oncology Laboratories, Weatherall Institute of Molecular Medicine, University of Oxford, John Radcliffe Hospital, Oxford, OX3 9DS UK; 2 Department of Oncology, Old Road Campus Research Building, University of Oxford, Oxford, OX3 7DQ UK; 3 Nuffield Department of Medicine, NDM Research Building, University of Oxford, Oxford, OX3 7DQ UK; 4 Department of Oncology, Molecular Oncology Group, Biodonostia Health Research Institute, Paseo Doctor Begiristain s/n, 20014 San-Sebastián, Spain

**Keywords:** HIF2α, Renal cancer, EMT, Drug resistance

## Abstract

**Background:**

In clear cell renal cell carcinoma, 80% of cases have biallelic inactivation of the *VHL* gene, leading to constitutive activation of both HIF1α and HIF2α. As HIF2α is the driver of the disease promoting tumour growth and metastasis, drugs targeting HIF2α have been developed. However, resistance is common, therefore new therapies are needed.

**Methods:**

We assessed the effect of the HIF2α antagonist PT2385 in several steps of tumour development and performed RNAseq to identify genes differentially expressed upon treatment. A drug screening was used to identify drugs with antiproliferative effects on *VHL-*mutated HIF2α-expressing cells and could increase effectiveness of PT2385.

**Results:**

PT2385 did not reduce cell proliferation or clonogenicity but, in contrast to the genetic silencing of HIF2α, it reduced in vitro cell invasion. Many HIF-inducible genes were down-regulated upon PT2385 treatment, whereas some genes involved in cell migration or extracellular matrix were up-regulated. HIF2α was associated with resistance to statins, addition to PT2385 did not increase the sensitivity. Conclusions: this study shows key differences between inhibiting a target versus knockdown, which are potentially targetable.

**Supplementary Information:**

The online version contains supplementary material available at 10.1186/s12885-021-08616-8.

## Background

Renal cell carcinoma (RCC) is amongst the 10 most common cancers [1]. The most common subtype of RCC is clear cell RCC (ccRCC, 70–85% of cases), characterised by high vascularity and showing lipid and glycogen accumulation in the cytoplasm [2]. Most ccRCC cases present biallelic inactivation of the von Hippel Lindau (*VHL*) tumour suppressor gene. Under normal oxygen conditions, VHL polyubiquitinates hypoxia-inducible factor 1 alpha (HIF1α) and 2 alpha (HIF2α) targeting them for proteasomal degradation, but in the absence of VHL, HIFα subunits can translocate to the nucleus, dimerize with HIF1β and transactivate the expression of their downstream genes [3]. Both HIF1α and HIF2α appear to be involved in ccRCC initiation, however, they have contrasting roles as the disease develops [4]. In contrast to other cancers, in ccRCC HIF1α functions as a tumour suppressor by attenuating tumour cell growth, whereas HIF2α promotes tumour development [4, 5]. This is partially achieved by the opposing effects on the oncogene MYC, with HIF2α enhancing MYC activity and the consequent alteration in the DNA methylation patterns, whereas HIF1α impairs MYC activity [6, 7]. Additionally, HIF pathway deregulation due to *VHL* mutation leads to ccRCC angiogenesis, and therefore, to the characteristic high vasculature of this tumour type [8]. Similarly, HIFs regulate every step of the metastatic process: from cell acquisition of motile and invasive phenotype (epithelial to mesenchymal transition, EMT), to inhibition of anoikis and later establishment of the premetastatic site prior to clonal expansion [9, 10]. Epigenetic alterations such as DNA methylation can regulate HIF2α-induced expression of metastatic genes in ccRCC [11], and superenhancer formation in inflammatory ccRCC cells promotes neutrophil-dependent lung metastasis [12]. Overall, HIF2α promotes metastasis in RCC [13, 14], and high HIF2α mRNA and protein levels in tumour tissue is associated with shorter survival [15].

Due to HIF2α involvement in ccRCC progression, drugs targeting HIF2α have recently been developed. Scheuermann et al. showed that small-molecule ligands such as PT2385, PT2399 and PT2977 can bind to a large hydrophobic cavity in the PAS-B domain of HIF2α, induce a conformational change, avoid the heterodimerization with HIF1β and finally impair the activation of downstream target gene expression [16–18]. PT2385 treatment inhibited the expression of HIF2α target genes in ccRCC cell lines and tumour xenografts and it promoted tumour regression faster than sunitinib [19], as did PT2399 [20]. In addition, PT2399 was demonstrated to reduce lung metastasis in animal models *in vivo* [21]. A phase I trial in previously treated patients showed that PT2385 was well tolerated and had a favourable safety profile, as no dose-limiting toxicities were observed at any dose level tested [22]. However, evaluation of the pharmacokinetic profiles of PT2385 showed that a significant proportion of patients were underexposed. These results promoted the development of the second-generation HIF2α antagonist PT2977 with the aim of improving PT2385’s variable and dose-limited pharmacokinetics resulting from extensive metabolism of PT2385 to its glucuronide metabolite [23].

Nevertheless, long term exposure to these HIF2α inhibitors generates resistance via mutations in the HIF2α binding pocket or in the heterodimerization partner HIF1β [20, 24]. Therefore, it is necessary to use a different approach to discover drugs against this malignancy. Statins (small-molecule inhibitors of the 3-hydroxy-3-methyl-glutaryl-coenzyme A (HMG-CoA) reductase (HMGR), the rate limiting enzyme of the mevalonate pathway) are reported to be differentially toxic for VHL-defective ccRCC cell lines [25], suggesting that repurposing well-known and well-characterized drugs could provide a novel therapeutic strategy to target ccRCC combined with PT2385, as statins have long been used to reduce cholesterol levels [26].

We investigated the effects of PT2385 in ccRCC by analysing migration, invasion, the clonogenic potential and the alteration in gene expression. In addition, we evaluated the effect of the drugs in the Pharmakon 1600 library to identify currently used or approved drugs with possible additive effects in the treatment of ccRCC.

## Methods

### Cell culture and cell transfection

Both 786–0 cells (obtained from the American Type Culture Collection (ATCC®), CRL-1932™) and RCC4 cells, gift from W. Kaelin [27], were cultured in DMEM low glucose medium (1 g/L) supplemented with 10% FBS no longer than 20 passages. They were mycoplasma tested every 3 months and all of them were authenticated using DNA STR analysis. 786–0 wild type (786–0 WT) cell line is *VHL* defective and contains an inactivating mutation in *HIF1α* gene, leading to constitutive expression of HIF2α. RCC4 *VHL* mutant cell line stably expressing an empty vector (RCC4 WT) or a vector for VHL overexpression (RCC4 VHL) were used (Table 1).

Transfection of HIF2α siRNA (siHIF2α) and siRNA control (siCON) (Supplementary Table 1) was performed with 12,000 cells using Optimem reduced serum medium at a final concentration of 20 nM. Oligofectamine (12252–011, Thermo Fisher) was used following the manufacturer’s instructions.

**Table 1:** Cell lines

Cell line	VHL expression	HIF1α expression	HIF2α expression	Genetic modification
786-0 WT	**-**	**-**	+	**-**
RCC4 WT	**-**	+	+	**-**
RCC4 VHL	+	**-**	**-**	VHL overexpression
786-0 siCON	**-**	**-**	+	siRNA control
786-0 siHIF2α	**-**	**-**	**-**	siRNA for HIF2α

### Western blot

Cells were washed with cold PBS and lysed 30 min on ice with RIPA lysis buffer (R0278, Sigma) containing protease (cOmplete, 11697498001, Roche) and phosphatase (phosSTOP, 4906845001, Sigma) inhibitor cocktails. Lysates were cleared by centrifugation and supernatants were boiled at 95 °C for 5 min in 4x NuPAGE LDS sample buffer (NP0007, Invitrogen) containing 10% β-mercaptoethanol. Samples were run on NuPAGE Novex 4–12% Bis-TRIS gels (NP0336BOX, Invitrogen) using NuPAGE MOPS-SDS running buffer (NP000102, Invitrogen) and afterwards, proteins were transferred to PVDF membranes (IPVH00010, Millipore). Membranes were blocked with 5% milk (A0830, Applichem) in TBS-T (TBS containing 0.1% Tween-20) for 1 h at room temperature and were then incubated overnight with anti-HIF2α primary antibody (NB100–122, Novus Biologicals) or β-actin peroxidase (A3854, Sigma) in 5% milk TBS-T at 4 °C. Membranes were washed 3x in TBS-T and incubated with HRP-antirabbit secondary antibody (P0448, Agilent). Development was performed with Amersham ECL Prime Western Blotting Detection Reagent (GERPN2232, Sigma) using ImageQuant™ LAS 4000.

### RT-qPCR

RNA was extracted using the Tri-Reagent protocol (T9424, Sigma) and 1 μg of RNA was reverse transcribed into cDNA with the High Capacity cDNA reverse transcription kit (44368813, Thermo Fisher) using random hexamer primers. The PCR reaction containing SensiMix™ SYBR Green® No-ROX Kit (QT650–20, Bioline) was run on a 7900 Real time PCR System with standard cycling conditions: 10 min 95 °C, and 40 cycles of 15 s 95 °C followed by 1 min 60 °C. Gene expression was analysed with the Ct method using *HPRT1* expression for normalization [28]. The primers used are listed in Supplementary Table 2.

### Migration assay

1.2 × 10^4^ 786–0 cells or 2 × 10^4^ RCC4 cells were seeded per well in 96-well ImageLock™ plates (4379, Essen BioScience) and incubated for 24 h. When the effect of PT2385 (B1920, BioVision) was tested, the compound was added to the wells once the cells were attached. After wounding, cells were washed with fresh media and plates were placed into the IncuCyte ZOOM® until wound closure. Scanning was performed using a 10x objective and scheduled every 2 h. Migration ability of the cells was analysed through two integrated metrics that the IncuCyte™ Software calculates based on the processed images: wound width and wound confluence. Wound width represents the average distance (μm) between the leading edge of the population of migrating cells (scratch wound mask) within an image. Wound confluence determines the percentage of wound area that is occupied by cells, and it relies on the initial scratch wound mask to differentiate the wounded from the non-wounded region.

### Invasion assay

96-well ImageLock™ plate wells were coated with a thin layer of Matrigel® Growth Factor Reduced Basement Membrane Matrix (354,230, Corning) and the plate was placed in a 37 °C incubator, 5% CO_2_ overnight. Matrigel® was removed and 1.2 × 10^4^ 786–0 cells or 2 × 10^4^ RCC4 cells were seeded per well and incubated for 24 h. PT2385 was added once the cells were attached. After performing the wound and washing the wells, 50 μL Matrigel® (8 mg/mL) was added and the plate was placed in the incubator for 30 min. After this time, 100 μL cell culture media (containing PT2385 in the corresponding experiment) was added to each well. The plate was then placed into the IncuCyte ZOOM® for 5 days. Scanning was performed using a 10x objective and scheduled every 4 h. The invasion ability of the cells was analysed using the relative wound density (RWD), which represents the density of the wound region relative to the density of the cell region, relying on the initial scratch wound mask to differentiate between cell-occupied and cell-free regions of the image.

### Cell proliferation assay

To validate the screening hits, simvastatin (S6196, Sigma), fluvastatin sodium hydrate (SML0038, Sigma) and terbinafine hydrochloride (T8826, Sigma) were added to the cell culture and cell viability was analysed 5 days later using CyQUANT™ Cell Proliferation Assay (C7026, Invitrogen) as per manufacturer’s instructions.

CyQUANT™ Cell Proliferation Assay was also used to determine cell proliferation after PT2385 treatment.

### Colony formation assay

1000 cells were plated in 100 mm plates in 20 mL media and cultured for 10 days. Media was removed and Coomassie blue solution (H_2_O containing 50% methanol, 7% acetic acid glacial (A/0360/PB17, Thermo Fisher) and 0.1% Brilliant Blue R (B7920, Sigma)) was added to fix and stain the colonies for 2 h. Then, Coomassie blue solution was recovered and plates were washed with tap water and allowed to dry overnight. Plates were scanned using UMAX MagicScan software. Colony count was manually performed using Fiji Image J Software.

### RNA sequencing (RNAseq)

The sequencing reads were checked for their quality using *FastQC* and low quality reads were trimmed using *cutadapt*. The trimmed reads were aligned to the human genome using *STAR*. Number of reads per gene (gene counts) was calculated using *featureCount*. Human assembly release GRCh38 was used for alignment and gene counting.

Differentially expressed genes (padj cut off < 0.05) and enriched gene ontological terms on biological processes or cellular components between 786-0 WT and 786–0 WT cells treated with 10 μM PT2385 for 48 h were identified and compared using the R package -*DESeq2* and *ClusterProfiler*, respectively. In short, three replicates of the experimental set were compared against three replicates of control set after removal of genes with very low counts.

The top 50 most up-regulated and 50 most down-regulated genes upon PT2385 treatment were extracted from the gene expression matrix after removing gene duplicates and used to create the heatmap.

### High throughput screening

300 786–0 WT cells per well were seeded in 384-well plates (GN781090, Sigma) using a Perkin Elmer FlexDrop reagent dispenser the day before treatment. The Pharmakon 1600 library (MicroSource Discovery Systems), including antibacterial, antidiabetic, antifungal, antihypertensive, anti-inflammatory, diuretic, histamine or neurotransmitter-related drugs, among others, was diluted and added to the cells using a Janus automated workstation (PerkinElmer) resulting in final concentrations of 10 μM, 1 μM and 0.1 μM, in duplicate. After a 3-day incubation the growth media was replaced with phenol red-free complete media containing 10 μg/mL resazurin. The plates were incubated at 37 °C, 5% CO_2_ for 2 h and then fluorescence was read using an Envision plate reader (PerkinElmer). After background subtraction, the data from each plate was normalised by calculating Z-scores. With the exception of a single plate, which was excluded from the analysis due to a plating error, the correlation between the replicates was good, with an average Pearson’s coefficient of 0.920.

### Statistical analysis

GraphPad Prism 5.0 statistical analysis software (GraphPad Software) was used. When analysing the influence of two different independent variables on one dependent variable, 2-way ANOVA with Bonferroni post hoc test was applied. When two means were compared, t-test was performed.

## Results

### PT2385 does not inhibit growth of ccRCC cells

The effect of the HIF2α analogue PT2385 was assessed in ccRCC cell proliferation and clonogenic survival. PT2385 did not alter 786–0 WT cell proliferation (Supplementary Figure 1A) nor colony formation (Supplementary Figure 1B). The RCC4 cell line was also analysed. RCC4 VHL cells (reconstituted with non-mutated *VHL*) generated more colonies than RCC4 WT cells (which express both HIF1α and HIF2α) and addition of PT2385 did not reduce the clonogenic potential of RCC4 WT cells (Supplementary Figure 1C), demonstrating that it does not inhibit in vitro cell growth*.*

### PT2385 treatment promotes tumour cell migration in vitro

**Fig. 1:**
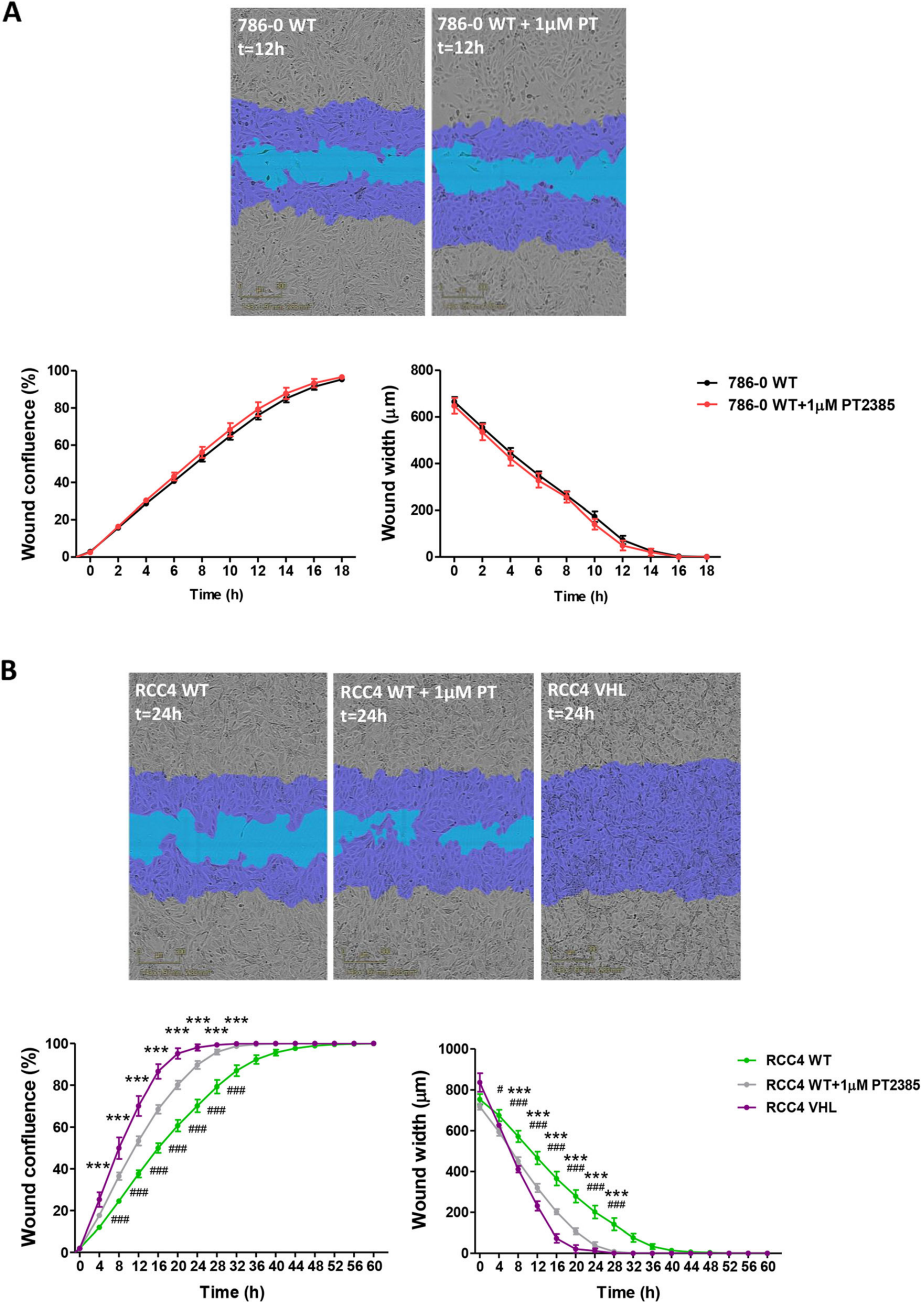
786–0 and RCC4 cell migration. **A**) Representative images of 786–0 WT and 786–0 WT + 1 μM PT2385 (PT) cells migrating 12 h after the scratch and wound confluence and wound width measure until the complete closure of the wound. n = 3. **B**) Representative images of RCC4 WT, RCC4 WT + 1 μM PT2385 (PT) and RCC4 VHL cells migrating 24 h after the scratch and wound confluence and wound width measure until the complete closure of the wound. n = 3. * represents comparison between RCC4 WT and RCC4 VHL and # represents comparison between RCC4 WT and RCC4 WT + 1 μM PT2385. Purple region corresponds to the initial scratch mask, whereas blue region represents the wound lacking cells at the compared moment. * *p* < 0.05, ** *p* < 0.01, *** *p* < 0.001. Errors bars depict standard error of the mean

The effect of PT2385 on 786–0 and RCC4 cell migration was analysed using the IncuCyte ZOOM®. Whereas PT2385 did not change 786–0 cell migration (Fig. 1A), it promoted the migration of RCC4 WT cells (Fig. 1B). The RCC4 VHL cell line migrated faster than RCC4 WT closing the wound 24 h after making the scratch compared to the 48 h needed by RCC4 WT cells (Fig. 1B). Interestingly, PT2385 addition to RCC4 WT cells promoted their migration generating an intermediate phenotype between RCC4 WT and RCC4 VHL cells (Fig. 1B). This suggests that in the RCC4 cell line, not only HIF2α but also HIF1α is repressing cell migration.

### HIF2α inhibition suppresses cell invasion in vitro

**Fig. 2:**
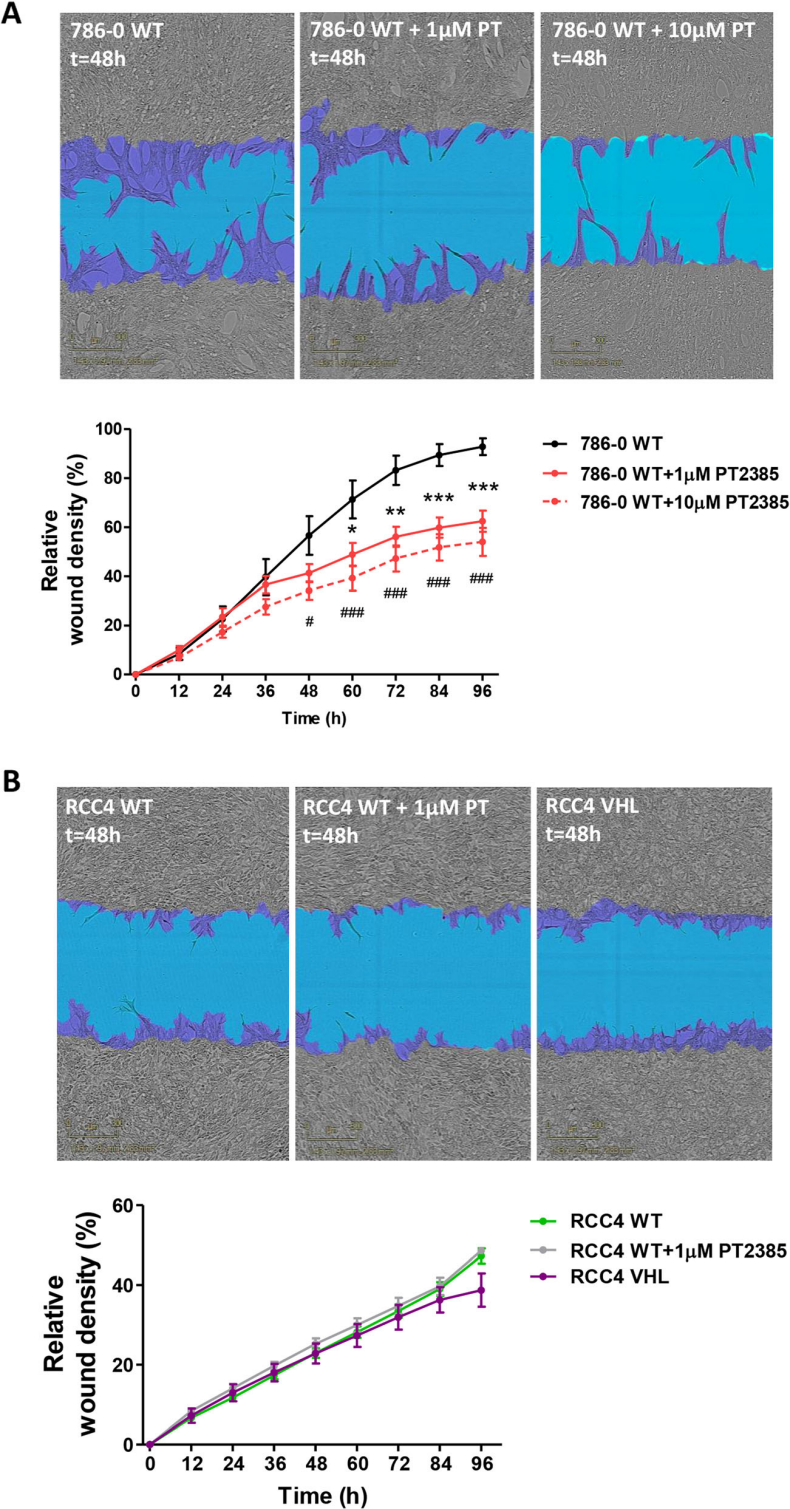
786–0 and RCC4 cell invasion. **A**) Representative images of 786–0 WT and 786–0 WT + 1/10 μM PT2385 (PT) cells invading 48 h after the scratch and RWD measurement until 96 h after wound performing. n = 3. * represents comparison between 786-0 WT and 786–0 WT + 1 μM PT2385 and # represents comparison between 786-0 WT and 786–0 WT + 10 μM PT2385. **B**) Representative images of RCC4 WT, RCC4 WT + 1 μM PT2385 (PT) and RCC4 VHL cells invading the wound 48 h after the scratch and RWD measurement until 96 h after wound performing. Purple region corresponds to the initial scratch mask, whereas blue region represents the wound lacking cells at the compared moment. n = 3. * *p* < 0.05, ** *p* < 0.01, *** *p* < 0.001. Errors bars depict standard error of the mean

The invasion ability of these cell lines was also evaluated using the IncuCyte ZOOM®. Addition of PT2385 impeded invasion of 786–0 WT cells in a concentration-dependent manner (Fig. 2A). Conversely, neither RCC4 WT nor RCC4 VHL cells were able to invade through the Matrigel®, and PT2385 treatment did not have any effect (Fig. 2B).

### HIF2α silencing does not affect cell migration or invasion 

786–0 WT cells were transfected with either siCON or siHIF2α and their migratory and invasion potential was analysed. Similarly to HIF2α inhibition using PT2385, suppression of HIF2α with siRNA did not alter the migratory ability of 786–0 WT cells (Supplementary Figure 2A), but in contrast to the inhibitor, HIF2α silencing did not reduce their invasion potential (Supplementary Figure 2A). To exclude a possible residual HIF2α effect in the observed phenotype of 786–0 WT siHIF2α cells, HIF2α protein levels and expression of HIF2α target genes was analysed every 24 h until the end of the experiment. HIF2α was not induced over time (Supplementary Figure 2B), and its downstream targets *GLUT1* and *VEGFA* were not consequently up-regulated (Supplementary Figure 2C).

### RNAseq comparison of the parental cell line with PT2385 effects

RNAseq showed that the most differentially expressed genes upon PT2385 treatment were related to renal development and hypoxia biological processes, followed by GO terms involved in cell migration, such as actin filament organization, tissue migration or regulation of cytoskeleton organization (Supplementary Figure 3A). Supporting the enriched GO terms for biological processes, enriched GO terms for cellular components were related to cell migration/invasion and cell-cell or cell-extracellular matrix interaction (ECM) (Supplementary Figure 3B).

**Fig. 3:**
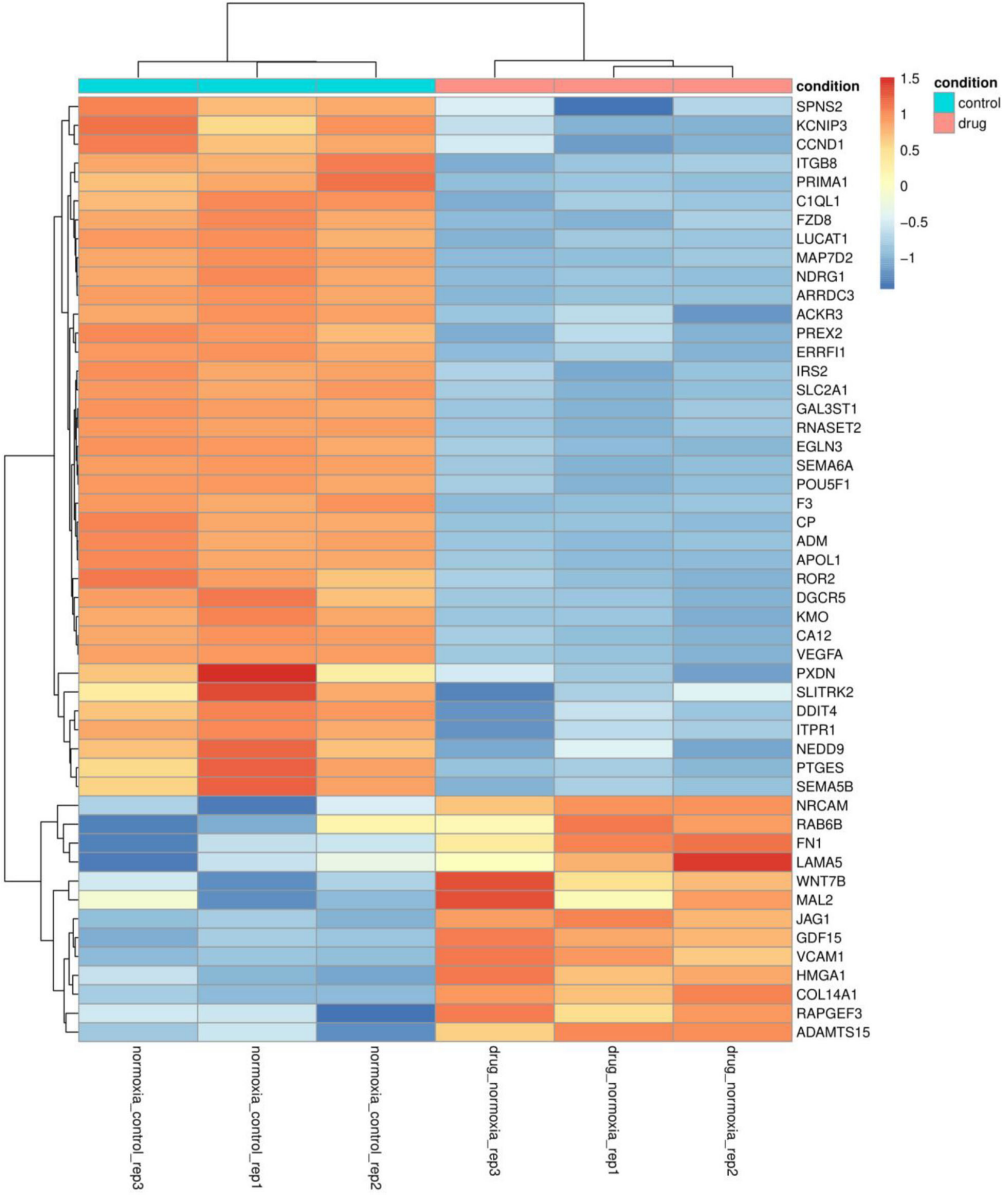
RNAseq results. Heatmap of the top 50 variant significant genes in 786–0 WT cells vs PT2385 treatment

Additionally, RNAseq analysis showed an expected down-regulation of many well documented HIF-induced genes upon PT2385 treatment (e.g. *NDRG1, SLC2A1, EGLN3* or *ROR2*) but several other genes were up-regulated (Fig. 3, see Supplementary Table 3  for full names). This last group included genes involved in cell migration and ECM (e.g. *RAB6B*, *FN1, VCAM1* or *COL14A1*) and genes of signalling pathways usually deregulated in cancer, such as Notch and Wnt signalling (*JAG1* and *WNT7B*, respectively).

### HIF2α confers resistance to statin treatment

The lack of cell growth inhibition by PT2385 but its effect on tumour cell movement led us to investigate if there were currently used drugs to which ccRCC cells would be sensitised.

**Fig. 4:**
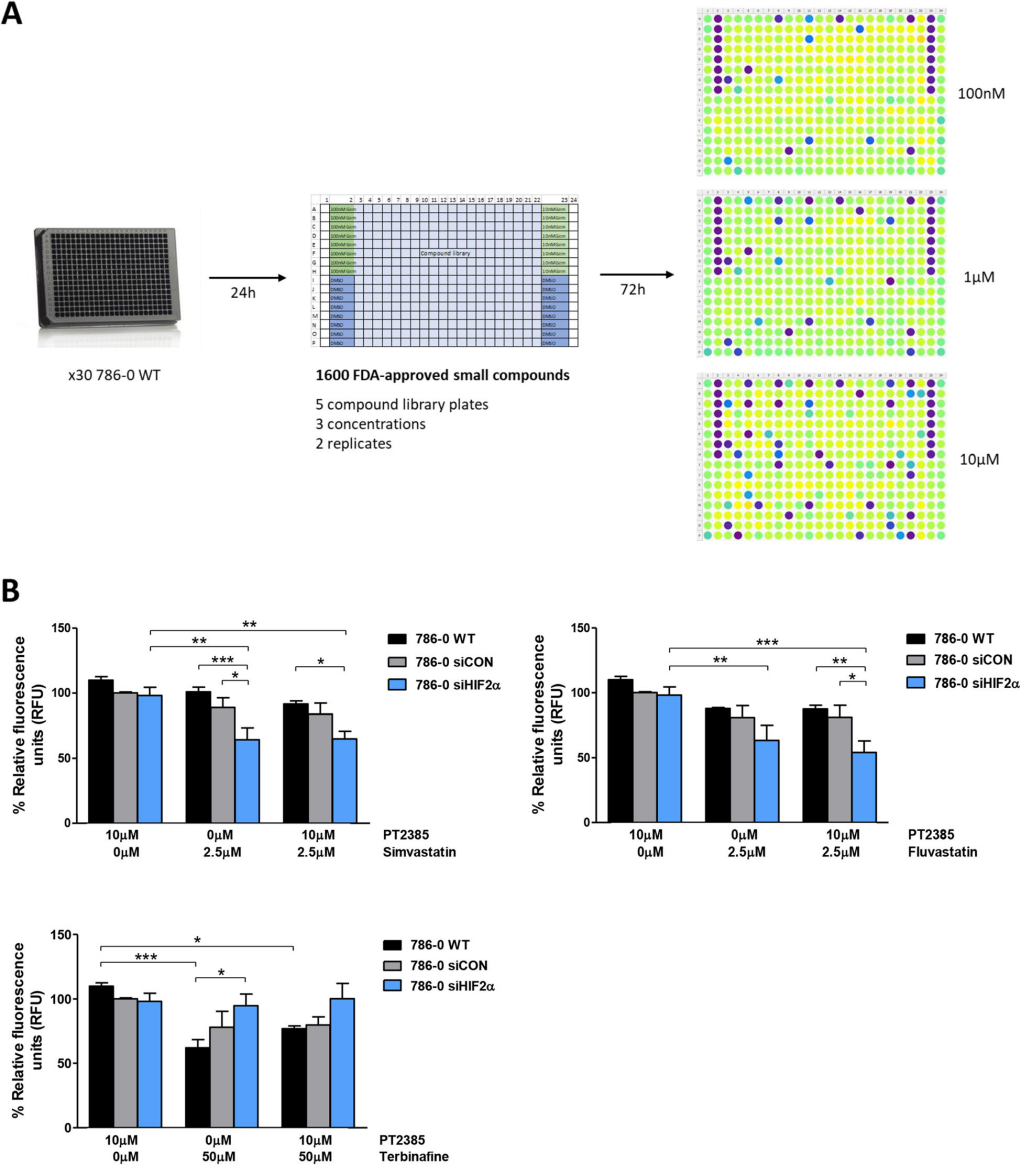
Drug screening of 786–0 WT cells. **A**) Diagram showing the drug screening. **B**) Viability of 786–0 WT and 786–0 WT cells transfected with siCON or siHIF2α and treated with 2.5 μM statins or 50 μM terbinafine +/− 10 μM PT2385 (PT) during 5 days relative to cells treated with DMSO. n = 3. * *p* < 0.05, ** *p* < 0.01, *** *p* < 0.001. Errors bars depict standard error of the mean

The drug screening performed in 786–0 WT cells identified several lethal compounds (Fig. 4A). Taking into account previously published data [25], the statins simvastatin and fluvastatin were further analysed, as well as the squalene monooxygenase inhibitor terbinafine, which blocks cholesterol synthesis while allowing the synthesis of non-sterol isoprenoids. We showed that the resistance of 786–0 WT cells to statins was due to HIF2α expression, as silencing HIF2α made the cells more sensitive (Fig. 4B). Supporting the no effect on viability, addition of the HIF2α antagonist did not contribute further to the antiproliferative effects of statins on 786–0 WT cells. However, HIF2α-expressing cells appeared to be more sensitive to terbinafine (Fig. 4B), and as for the statins, addition of PT2385 did not have any additional effect.

## Discussion

Metastasis is a multistep process which selects for highly aggressive tumour cells, as they acquire the ability to disseminate from the primary tumour and grow at distant sites [29]. Here, we show that the oncoprotein HIF2α is involved in in vitro cell migration and invasion in ccRCC (Table 2), as has already been described for many tumour cell lines [30–32].

**Table 2:** Results summary

Assay	Cell line	Genetic modification / Treatment	Results compared to control
Proliferation	786-0 WT	PT2385	=
Clonogenic assay	786-0 WT	PT2385	=
	RCC4 VHL	VHL overexpression	+
	RCC4 WT	PT2385	=
Migration	786-0 WT	PT2385	=
	786-0 WT	siHIF2α	=
	RCC4 WT	PT2385	+
	RCC4 VHL	VHL overexpression	+
Invasion	786-0 WT	PT2385	**-**
	RCC4 VHL	VHL overexpression	=
	RCC4 WT	PT2385	=
Sensitivity to statins	786-0 WT	siHIF2α	+
Sensitivity to terbinafine	786-0 WT	siHIF2α	**-**

RCC4 VHL results are compared to RCC4 WT cells

786-0 siHIF2α results are compared to 786-0 siCON cells

^a^PT2385 treatment results are compared to non-treated cells

As previously reported [19], the HIF2α antagonist PT2385 did not affect ccRCC cell proliferation nor colony formation. Interestingly, HIF2α blockage by PT2385 or siRNA had opposing effects. Treatment with PT2385 did not affect 786–0 cell migration, while partially abolishing cell invasion in a concentration-dependent manner. However, silencing of HIF2α by siRNA did not change the migration or invasion ability of 786–0 cells. PT2385 allosterically binds to HIF2α and thereby prevents the heterodimerization with HIF1β and its subsequent binding to the DNA [33], whereas siRNA binds to complementary mRNA and targets them for degradation in a transitory manner [34]. HIF2α silencing was successfully achieved during the invasion experiment but at the endpoint (96 h), HIF2α started to re-express. These results suggest that the few molecules escaping siRNA silencing might be interacting with other molecules, e.g. the MYC/MAX complex [6, 7], to keep the phenotype, even though they are not detectable at protein level. On the other hand, PT2385 treatment showed that DNA binding is not completely abolished by the inhibitor, as previously reported [24], as cell invasion was not 100% suppressed.

Supporting the role of HIF2α in the regulation of ccRCC migration, RCC4 VHL cells migrated more than RCC4 WT, in contrast to previous publications [35, 36]. In this case, as RCC4 WT cells also express HIF1α, both HIF2α and HIF1α could be inhibiting cell migration; however, treatment with PT2385 generated an intermediate phenotype pointing to a more important role of HIF2α.

RNAseq results showed the already known specificity of PT2385 for HIF2α. PT2385 treatment down-regulated the expression of genes involved in hypoxic response (*EGLN3* or *CA12*), migration (*SEMA6A/5B*) and metastasis (*ITGB8* or *VEGFA*). These results support the previously described effect of PT2385 avoiding ccRCC tumour progression and metastasis [19]. On the other hand, PT2385 treatment increased the expression of genes involved in cell-cell or cell-ECM interaction, such as *FN1, VCAM1, COL14A1* or *ADAMTS15.* High abundance of components of the ECM like fibronectin 1 or collagen, provided by Matrigel® in our experiments, can possibly explain the inhibition of PT2385 in cell invasion, as the cells might not be able to degrade the ECM and move through it. In addition, high levels of cell-cell adhesion molecules such as VCAM1 could also reduce cell movement.

However, the increased expression of genes upon PT2385 treatment suggests that one way of enhancing the effect of PT2385 could be via combination therapy targeting those molecules. Fibronectin, for instance, exists in multiple isoforms and in adulthood the expression of EDA and EDB domains is very restricted in normal tissue, whereas it is highly expressed in tumours [37]. This has led to the development of drugs or antibodies against these domains as a mechanism of delivering drugs to the tumour site [38, 39]. Treatment of PT2385 increases FN1 expression, increasing the amount of target fibronectin in the tumour and possibly making it easier to specifically deliver tumour-directed drugs. In addition, PT2385 treatment increased the expression of JAG1 in 786–0 WT cells, suggesting that combining Notch signalling inhibitors already used in clinic with PT2385 could be of benefit for renal cancer treatment. Bhagat et al. (2016) found that genetic and epigenetic alterations in ccRCC tissues led to both Notch ligand and receptor overexpression [40]. JAG1, for instance, was overexpressed and associated with loss of CpG methylation of HeK4me1-associated enhancer regions. They confirmed the procarcinogenic role of Notch in vivo, as previously reported [41], and showed that treatment with the gamma-secretase inhibitor LY3039478 avoided ccRCC cell growth both in vitro and in vivo.

Previous reports demonstrated that HIF2α silencing does not affect in vitro ccRCC growth under standard culture conditions [4, 42]. Similarly, we showed that addition of PT2385 did not inhibit tumour cell proliferation or colony formation at concentrations up to 10 μM, and its combination with statins did not further contribute to the antiproliferative effects of statins. Both the synthetic statin fluvastatin and the semi-synthetic statin simvastatin impaired proliferation in HIF2α knockdown cells.

Another approach for developing new therapy options in combination with PT2385 would be to identify target genes with synthetic lethal relationship with HIF2α silencing. Nicholson et al. identified CDK4 and CDK6 as genes with lethal relationship with *VHL* loss, as loss of either gene alone was well tolerated, but the concurrent loss of both was lethal [43]. Supporting our results, they found that both simvastatin and fluvastatin inhibited the growth of VHL-reconstituted 786–0 cells more substantially than their VHL-defective counterparts. However, in contrast to a previous study [25], our results showed that statin-induced lethality is not due to *VHL* loss and the consequent HIFs expression, but associated with HIF2α loss. Thus HIF2α-conferred protection against statins suggests that one way of repurposing these drugs could be via combination treatment with HIF2α antagonists. Although we were not able to detect differences in cell proliferation in vitro, previously published data on HIF2α antagonists showed in vivo effects [19, 20]. However, we are in agreement with Thompson et al. [25] suggesting that the key branch for the observed phenotype is the blockage of isoprenylation and not the cholesterol synthesis pathway, as the lethal effect could not be rescued after treatment with squalene [25] and 786–0 siHIF2α cells were not sensitive to terbinafine.

## Conclusions

Our study shows new therapy avenues to build on PT2385, as some of the genes that are up-regulated by HIF2α inhibition, are potential targets for combination treatments.

## Supplementary Information

**Additional file 1.**

**Additional file 2.**

**Additional file 3.**

**Additional file 4.**

**Additional file 5.**

## Data Availability

The RNAseq data generated and analysed during the current study is available in the NCBI’s Gene Expression Omnibus repository, and is accessible through GEO Series accession number GSE153711 (https://www.ncbi.nlm.nih.gov/geo/query/acc.cgi?acc=GSE153711).

## Abbreviations

ATCC: American Type Culture Collection
RCC: Renal cell carcinoma
ccRCC: Clear cell renal cell carcinoma
VHL: Von Hippel-Lindau
HIF1α: Hypoxia-inducible factor 1 alpha
HIF2α: Hypoxia-inducible factor 2 alpha
ECM: Extracellular matrix
EMT: Epithelial to mesenchymal transition
HMG-CoA: 3-hydroxy-3-methyl-glutaryl-coenzyme A
HMGR: HMG-CoA reductase
RWD: Relative wound density
padj: adjusted *p*-value
RNA sequencing: RNAseq
