# Supplementary material for: Differential effects of HIF2α antagonist and HIF2α silencing in renal cancer and sensitivity to repurposed drugs
Source: BMC Cancer. 2021 Aug 5;21:896. doi: 10.1186/s12885-021-08616-8 (PMC8344147; doi:10.1186/s12885-021-08616-8)
Supplement: Supplementary file 1 — Additional file 1. [file 12885_2021_8616_MOESM1_ESM.docx]

**Title: Differential effects of HIF2α antagonist and HIF2α silencing in renal cancer and sensitivity to repurposed drugs**

Esther Arnaiz^a^, Ana Miar^b‡^, Esther Bridges^a^, Naveen Prasad^b^, Stephanie B. Hatch^c^, Daniel Ebner^c^, Charles H. Lawrie^d*^, Adrian L. Harris^a*^

^a^ Department of Medical Oncology, Molecular Oncology Laboratories, Weatherall Institute of Molecular Medicine, University of Oxford, John Radcliffe Hospital, Oxford, OX3 9DS, UK

^b^ Department of Oncology, Old Road Campus Research Building, University of Oxford, Oxford, OX3 7DQ, UK

^c^ Nuffield Department of Medicine, NDM Research Building, University of Oxford, Oxford, OX3 7DQ, UK

^d^ Department of Oncology, Molecular Oncology Group, Biodonostia Health Research Institute, Paseo Doctor Begiristain s/n San-Sebastián, 20014, Spain

*These authors contributed equally to this work.

^‡^To whom correspondence should be addressed: Dr Ana Miar, Department of Oncology, Old Road Campus Research Building, University of Oxford, Oxford, OX3 7DQ, UK, e-mail: anbelen_mc@hotmail.com. Phone: +44 (0)1865 617318.

**SUPPLEMENTARY INFORMATION**

**Supplementary Table 1.** **Control (siCON) and HIF2α (siHIF2α) siRNA sequences.**

| **siRNA** | **Sequence (5’-3’)** |
| --- | --- |
| siCON | ACGACACGCAGGUCGUCAUTT* |
|  | TAACGACCTGAAGATTGAATT* |
| siHIF2α | CAAGCCACTGAGCGCAAATTT* |
|  | TGAATTCTACCATGCGCTATT* |

siHIF2α is a pool of three siRNA. TT* overhang.

**Supplementary Table 2. qPCR primers.**

| **Primer** | **Sequence (5’-3’)** |
| --- | --- |
| HPRT1_F | TGACACTGGCAAAACAATGCA |
| HPRT1_R | GGTCCTTTTCACCAGCAAGCT |
| HIF2α_F | CAACAGAGGCCGTACTGTCA |
| HIF2α_R | CACATGATGATGAGGCAGGA |
| VEGFA_F | CCTCCGAAACCATGAACTTT |
| VEGFA_R | ATGATTCTGCCCTCCTCCTT |
| GLUT1_F | GGTTGTGCCATACTCATGACC |
| GLUT1_R | CAGATAGGACATCCAGGGTAGC |

**Supplementary Table 3. Full name of the heatmap genes.**

| **Symbol** | **Full name** |
| --- | --- |
| SPNS2 | sphingolipid transporter 2 |
| KCNIP3 | potassium voltage-gated channel interacting protein 3 |
| CCND1 | cyclin D1 |
| ITGB8 | integrin subunit beta 8 |
| PRIMA1 | proline rich membrane anchor 1 |
| C1QL1 | complement C1q like 1 |
| FZD8 | frizzled class receptor 8 |
| LUCAT1 | lung cancer associated transcript 1 |
| MAP7D2 | MAP7 domain containing 2 |
| NDRG1 | N-myc downstream regulated 1 |
| ARRDC3 | arrestin domain containing 3 |
| ACKR3 | atypical chemokine receptor 3 |
| PREX2 | phosphatidylinositol-3,4,5-trisphosphate dependent Rac exchange factor 2 |
| ERRFI1 | ERBB receptor feedback inhibitor 1 |
| IRS2 | insulin receptor substrate 2 |
| SLC2A1 | solute carrier family 2 member 1 |
| GAL3ST1 | galactose-3-O-sulfotransferase 1 |
| RNASET2 | ribonuclease T2 |
| EGLN3 | egl-9 family hypoxia inducible factor 3 |
| SEMA6A | semaphorin 6A |
| POU5F1 | POU class 5 homeobox 1 |
| F3 | coagulation factor III, tissue factor |
| CP | ceruloplasmin |
| ADM | adrenomedullin |
| APOL1 | apolipoprotein L1 |
| ROR2 | receptor tyrosine kinase like orphan receptor 2 |
| DGCR5 | DiGeorge syndrome critical region gene 5 |
| KMO | kynurenine 3-monooxygenase |
| CA12 | carbonic anhydrase 12 |
| VEGFA | vascular endothelial growth factor A |
| PXDN | peroxidasin |
| SLITRK2 | SLIT and NTRK like family member 2 |
| DDIT4 | DNA damage inducible transcript 4 |
| ITPR1 | inositol 1,4,5-trisphosphate receptor type 1 |
| NEDD9 | neural precursor cell expressed, developmentally down-regulated 9 |
| PTGES | prostaglandin E synthase |
| SEMA5B | semaphorin 5B |
| NRCAM | neuronal cell adhesion molecule |
| RAB6B | RAB6B, member RAS oncogene family |
| FN1 | fibronectin 1 |
| LAMA5 | laminin subunit alpha 5 |
| WNT7B | Wnt family member 7B |
| MAL2 | mal, T cell differentiation protein 2 |
| JAG1 | jagged canonical Notch ligand 1 |
| GDF15 | growth differentiation factor 15 |
| VCAM1 | vascular cell adhesion molecule 1 |
| HMGA1 | high mobility group AT-hook 1 |
| COL14A1 | collagen type XIV alpha 1 chain |
| RAPGEF3 | Rap guanine nucleotide exchange factor 3 |
| ADAMTS15 | ADAM metallopeptidase with thrombospondin type 1 motif 15 |
