# Supplementary figures and images for: Differential effects of HIF2α antagonist and HIF2α silencing in renal cancer and sensitivity to repurposed drugs
Source: BMC Cancer. 2021 Aug 5;21:896. doi: 10.1186/s12885-021-08616-8 (PMC8344147; doi:10.1186/s12885-021-08616-8)

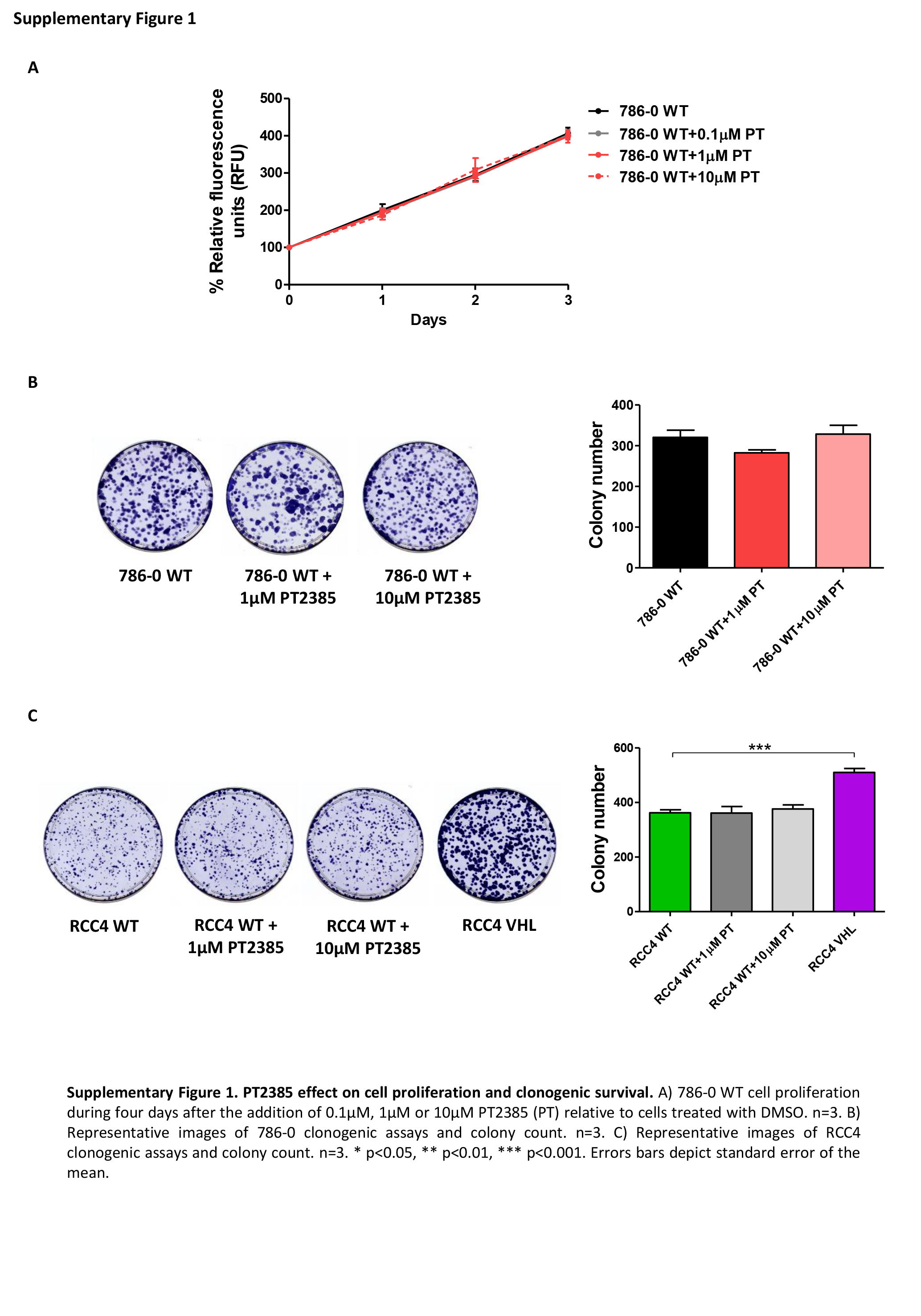

Supplement: Supplementary file 2 — Additional file 2. [file 12885_2021_8616_MOESM2_ESM.tif]

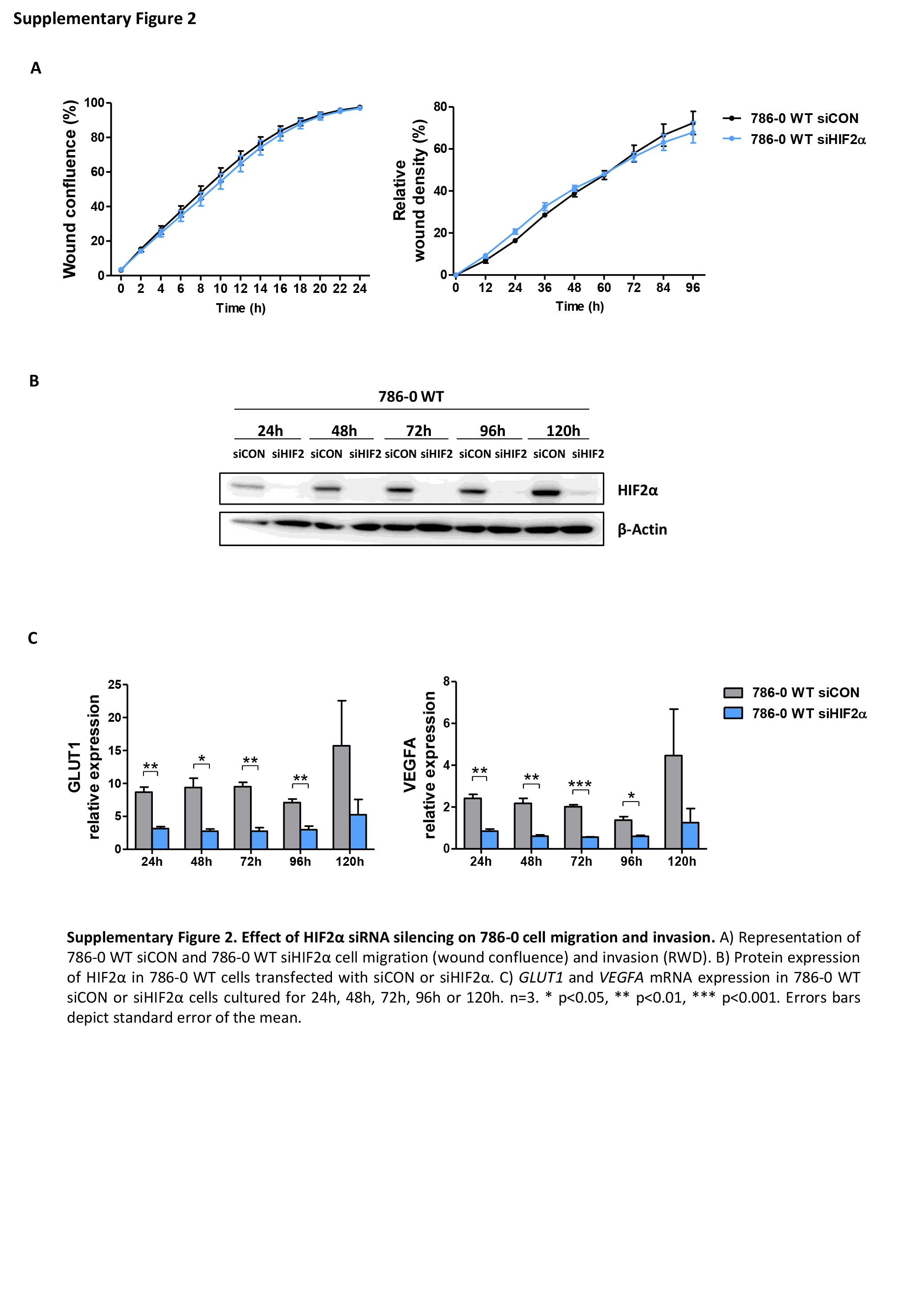

Supplement: Supplementary file 3 — Additional file 3. [file 12885_2021_8616_MOESM3_ESM.tif]

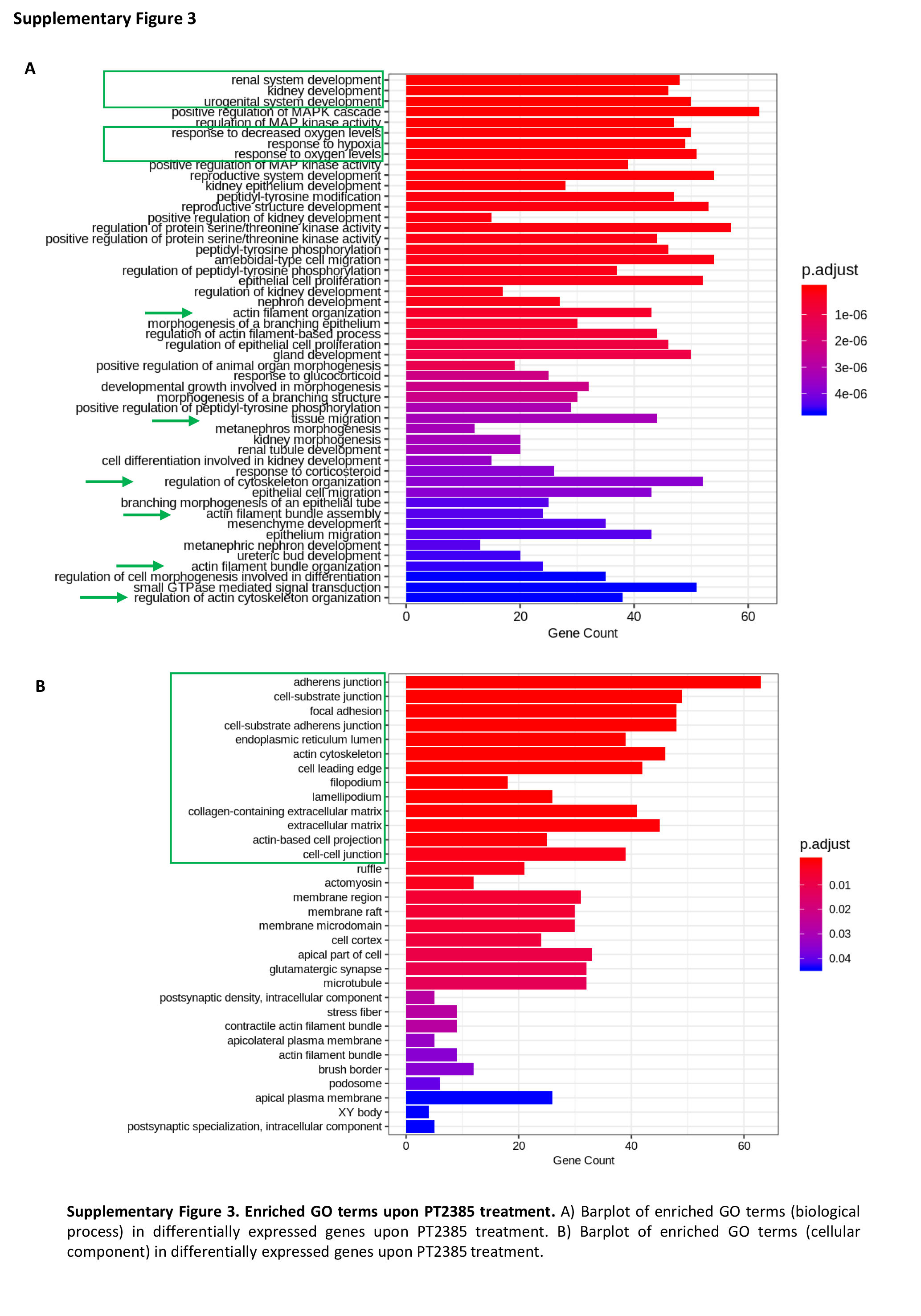

Supplement: Supplementary file 4 — Additional file 4. [file 12885_2021_8616_MOESM4_ESM.tif]
