# Supplementary material for: Differential effects of HIF2α antagonist and HIF2α silencing in renal cancer and sensitivity to repurposed drugs
Source: BMC Cancer. 2021 Aug 5;21:896. doi: 10.1186/s12885-021-08616-8 (PMC8344147; doi:10.1186/s12885-021-08616-8)
Supplement: Supplementary file 5 — Additional file 5. [file 12885_2021_8616_MOESM5_ESM.pdf]

Supplementary Figure 4

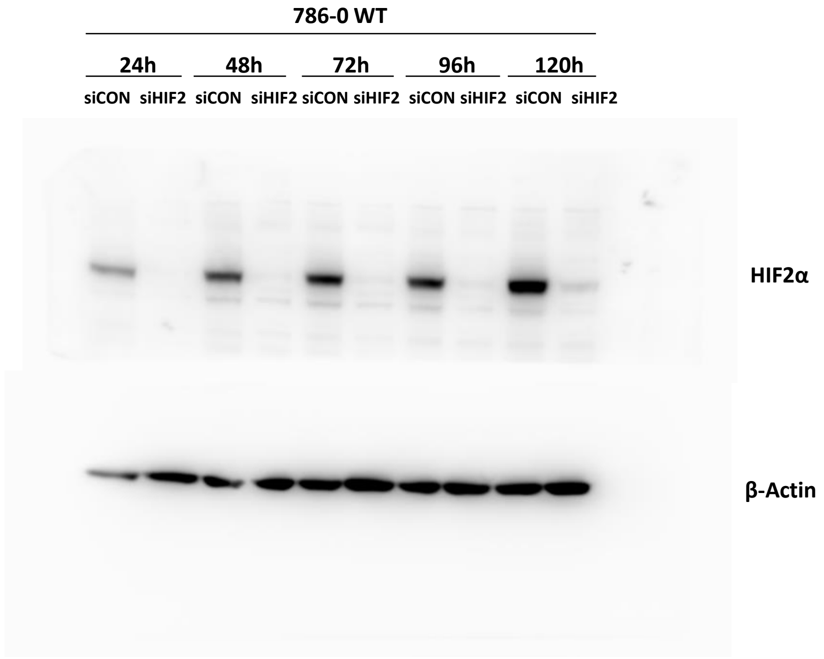

Supplementary Figure 4. Uncropped western blot. Protein expression of HIF2α in 786-0 WT cells transfected with siCON or siHIF2α and cultured for 24h, 48h, 72h, 96h or 120h. n=3.
